# Unexpected effects of long-term treatment with acetylsalicylic acid on late phase of pulmonary metastasis in murine model of orthotopic breast cancer

**DOI:** 10.1371/journal.pone.0230520

**Published:** 2020-04-06

**Authors:** Marta Smeda, Agnieszka Kij, Bartosz Proniewski, Karolina Matyjaszczyk-Gwarda, Kamil Przyborowski, Agnieszka Jasztal, Katarzyna Derszniak, Piotr Berkowicz, Anna Kieronska-Rudek, Marta Stojak, Magdalena Sternak, Stefan Chlopicki

**Affiliations:** 1 Jagiellonian Centre for Experimental Therapeutics, Jagiellonian University, Krakow, Poland; 2 Department of Pharmacology, Jagiellonian University Medical College, Krakow, Poland; Cleveland Clinic Lerner Research Institute, UNITED STATES

## Abstract

Long-term administration of acetylsalicylic acid (ASA) was effective in prevention of colorectal cancer, whereas the efficacy of this compound in other cancer types, including breast cancer, has been less convincingly documented. Indeed, the antimetastatic effect of low-dose ASA was observed only in the early intravascular phase of metastasis of breast cancer. In the present work, we characterized the effects of long-term treatment with ASA on the late phase of pulmonary metastasis in a mouse orthotopic 4T1 breast cancer model. Mice were treated with ASA at a dose of 12 mg·kg^-1^ of body weight daily starting one week prior to inoculation of 4T1 breast cancer cells, and the treatment was continued throughout progression of the disease. ASA administration decreased platelet TXB_2_ production in *ex vivo* assays but did not change thrombin-induced platelet reactivity. Although the number of metastases in the lungs remained unchanged in ASA-treated mice, infiltration of inflammatory cells was increased concomitantly with higher G-CSF and serotonin concentrations in the lungs. Pulmonary NO production was compromised compared to control 4T1 mice. ASA treatment also evoked an increase in platelet and granulocyte counts and decreased systemic NO bioavailability along with increased markers of systemic oxidant stress such as higher GSSG/lower GSH concentrations in RBC. Analysis of eicosanoids in stirred blood demonstrated that administration of ASA at a dose of 12 mg·kg^-1^ to cancer-bearing mice had an effect beyond inhibition of platelet COX-1, suggesting long-term treatment with low-dose aspirin is not a selective murine platelet COX-1/TXA_2_ pathway inhibitor in cancer-bearing mice. In summary, quite surprisingly, long-term treatment with low-dose ASA administered until the advanced phase of breast cancer in a murine orthotopic model of 4T1 breast cancer negatively affected the phenotype of the disease.

## Introduction

Acetylsalicylic acid (ASA) has been intensively studied over the last few decades with respect to its anti-metastatic effects. While at high doses ASA inhibits both isoforms of cyclooxygenase (COX-1 and COX-2) and has anti-proliferative/pro-apoptotic effects with respect to cancer cells, anti-metastatic effects of lower doses of ASA (≤100 mg·kg^-1^ per day in humans equivalent to ≤15 mg·kg^-1^ in mice [1]) are attributed to the inhibition of COX-1 and thromboxane A_2_ (TXA_2_) synthesis by platelets [2]. Indeed, a vast number of platelet-dependent mechanisms involved in cancer progression and metastasis have been identified [3]. Furthermore, in case studies and metanalysis of randomized clinical trials (where effects of ASA were examined in entities not related to cancer), it was documented that ASA used at low anti-platelet dose reduced primary cancer and metastasis incidence [4–7], however the effects were not confirmed in the elderly people [8]. Surprisingly, higher all-cause mortality was observed among initially healthy older adults who received daily aspirin than among those who received placebo and it was attributed primarily to cancer-related death [8]. Moreover, in particular types of cancer, the positive effects of ASA were also not so evident [9]. While ASA consistently lowered the incidence of gastrointestinal cancers (i.e. colorectal) [4] and is now recommended for primary prevention of colorectal cancer in adults older than 76 years [10], the evidence for the efficacy of ASA in breast cancer patients is less convincing, with some reports supporting the anti-metastatic activity [11], and others showing no evident reduction of both the risk [12, 13] and the number of deaths [6]. Therefore, there is an on-going ADD-ASPIRIN Trial [14] devoted to the potential use of ASA as an agent inhibiting post-surgery metastasis in cancer (including breast) patients, that will provide an important re-evaluation of and the anti-metastatic effects of aspirin, but the results have not yet been published.

In this context, many questions still need to be answered including the dosage, duration of use and optimal timing for initiation of the therapy with ASA to achieve the anticancer effects in various types of malignancies, in particular non-gastrointestinal ones [11]. Indeed, it has been recently shown that ASA-dependent inhibition of COX-1/TXA_2_ pathway in platelets effectively lowered metastatic spread of breast cancer only when applied in the early phase during formation of an intravascular metastatic niche, but anti-metastatic effects of the same dose were not observed when ASA was administered during the extravascular phase of metastasis [15].

To characterise the therapeutic efficacy of long-term treatment of ASA in breast cancer, we studied the effects of low-dose ASA (12 mg·kg^-1^) on disease progression and metastasis in an orthotopic murine model of breast cancer when therapy was initiated seven days prior to inoculation of 4T1 breast cancer cells, and continued throughout the progression of the disease, until its advanced stage. Our results clearly demonstrated that chronic use of ASA at such a dose and schedule of administration negatively affected the phenotype of the disease.

## Materials and methods

### Animals

Female BALB/c mice (aged 7–11 weeks) were purchased from the Medical University of Bialystok (Poland) and were housed 5–6 per cage, in a temperature-controlled environment (22–25°C), 12-hour light/day cycle and unlimited access to food (Zoolab, Krakow, Poland) and water throughout the experiment.

ASA administration to breast cancer-bearing mice. Fifty mice were randomly divided into control non-ASA-treated (4T1 group, 25 mice) and mice treated with ASA (4T1+ASA, 25 mice) (acetylsalicylic acid (Sigma Aldrich, A5376) premixed with chow) at a dose of ~12 mg per kg of body weight per 24 h that is equivalent to ~100 mg per 24 h in a 70 kg-human [1]. ASA treatment was started seven days prior to orthotopic inoculation of 4T1 breast cancer cells. All mice from control 4T1 and 4T1+ASA groups were orthotopically inoculated with 1x10^4^ of 4T1-luc2td-Tomato cells and all animals were euthanised in the 5^th^ terminal week of the disease progression by i.p. injection of ketamine and xylazine, 100 and 10 mg·kg^-1^, respectively. All experimental procedures involving animals performed to obtain the data presented in this study were specifically accepted by the Second Local Ethical Committee on Animal Testing in the Institute of Pharmacology, Polish Academy of Sciences (Krakow, Poland), permit no: 97/2016. Since orthotopic inoculation of cancer cells into the mammary fat pad of mice was not associated with animal distress higher than performing a subcutaneous injection (cancer cells were introduced in 50μl suspension by subcutaneous injection in the mammary fat pad), no anaesthesia was administered during this procedure according to the approval of the Second Local Ethical Committee on Animal Testing. Mice welfare was monitored throughout the study once daily and mice were euthanised (i.p. injection of ketamine and xylazine, 100 and 10 mg·kg^-1^, respectively) in case of evident (1) isolation of the animal from the group, and (2) evident (>20%) body weight loss.

### Cell culture

The mouse mammary adenocarcinoma 4T1-luc2-tdTomato cell line (in short: 4T1 cells) stably expressing the firefly luciferase gene and tdTomato fluorescent protein was a kind gift of Professor Joanna Wietrzyk (Ludwik Hirszfeld Institute of Immunology and Experimental Therapy, Polish Academy of Sciences) in 2015 at the 5^th^ passage from resuscitation after their purchase from Caliper Life Sciences Inc. (USA)) (the source of the parental line: ATCC, CRL-2539). The 4T1 cells were authenticated by Caliper Life Sciences Inc. by measurement of luciferase and tdTomato expression using an IVIS Spectrum Bioluminescence (BLI) and Fluorescence Activity (FLI). 4T1-luc2-tdTomato cells were cultured in RPMI1640 GlutaMAX Medium (Gibco) supplemented with 10% FBS (Gibco), antibiotic antimycotic solution, (AAS, Sigma-Aldrich) containing 20 units of penicillin, 20 mg of streptomycin and 0.05 mg of amphotericin B. Cells were cultured at 37°C in an atmosphere of 5% CO_2_ as previously described [16]. Prior to transplantations, cells were detached using Accutase solution (Sigma-Aldrich, Poland), centrifuged (300 x g, 4°C, 5 min), counted, suspended in Hank’s Balanced Salt Solution (HBSS, IIET, Poland) and inoculated into the mammary gland of female BALB/C mice. All cell cultures were routinely tested for *Mycoplasma* contamination.

### Blood collection

Blood was collected from the right heart ventricle on 3.8% citrate (9:1 v/v) and used for measurement of blood count, eicosanoid profile and platelet reactivity or centrifuged at 4°C at 1000 x g for 10 min to obtain plasma that was aliquoted and frozen at -80°C for subsequent analysis.

### Growth of primary tumour, pulmonary metastasis and lung airness

After euthanasia, primary tumours and lungs were excised and weighed. Lungs were fixed in 4% buffered formalin solution to assess pulmonary metastasis. The number of secondary nodules in the lungs was counted on the surface of lung lobes under a magnifying glass and on lung cross-sections stained with haematoxilin and eosin (H&E). Concomitantly, the relative metastatic area was measured and presented as the percentage of the cross-section area of the lung lobes. For assessment of relative lung nuclei area, ten randomly chosen eye fields of H&E stained lungs cross-sections were photographed by a blinded investigator with the exclusion of lung metastases as well as larger lung alveoli and segmented in *Ilastik* (developed by the Ilastik team, with partial financial support by the Heidelberg Collaboratory for Image Processing, HHMI Janelia Farm Research Campus and Cell Networks Excellence Cluster). The relative number of pixels corresponding to the area of the lung cross-sections occupied by the capillaries and other cells and nuclei of the cells in the lung parenchyma were counted in each experimental group using Image J [17].

### NO production

Colloidal Fe(DETC)_2_ was used for trapping the intracellular NO production in the lungs with EPR detection as described previously in mouse models of 4T1 breast cancer [16] and endotoxemia [18]. Briefly, lungs were perfused with ice-cold PBS, excised and cut into small pieces and incubated for 90 minutes at 37°C in colloidal Fe(DETC)_2_ in Krebs-HEPES buffer. Measurement of Fe(DETC)_2_-NO signal in frozen samples was performed in a finger Dewar using an EMX Plus Bruker spectrometer and the NO triplet amplitude normalised to wet tissue weight.

### Measurement of GSH and GSSG concentration by capillary electrophoresis

#### Capillary electrophoresis

A P/ACE MDQ capillary electrophoresis system (Beckman Coulter, Fullerton, CA, USA) with 32 Karat software (ver. 8.0, Beckman Coulter, Fullerton, CA, USA) was used for analyses. The apparatus was equipped with a PDA detector set at λ = 200 nm. Separation of the analytes took place in an uncoated fused-silica capillary (60.2 cm total length, 50 cm effective length, 50 μm i.d. and 375 μm o.d.) thermostated at 25°C with a constant voltage of 25 kV (~6.5 μA). BisTRIS (75 mmol/L), boric acid (25 mmol/L) buffer adjusted to pH 7.8 by adding 1 mol/L NaOH, was chosen as a background electrolyte (BGE). Samples were introduced to the capillary by hydrodynamic injection for 20 sec by 3.5 kPa, followed by injection of dd H_2_O for 2 sec by 3.5 kPa. Between analytical runs, the capillary was rinsed with 1 mol/L NaOH, deionised water, and BGE, respectively (138 kPa; 2 min each). Obtained data were analysed by PeakFit software (ver. 4.12, Systat software, San Jose, CA, USA).

#### Sample preparation

Measurements of GSSG and GSH were performed as described by [19]. Briefly, red blood cells (RBCs) were first separated from plasma by centrifuging whole blood. A hemolysate was prepared by adding to 100 μl of RBC, 400 μl of haemolysing reagent (10 mmol/L KCN and 5 mmol/L EDTA in dd H_2_O). Then, samples were deproteinised by adding 100 μl of 5% metaphosphoric acid (MPA) to 100 μl of hemolysate. After centrifugation (10,000 × g for 10 min at 4°C), the MPA extracts were diluted 1:4 with dd H_2_O. The calibration curves for GSH (2.5–80 μmol/L) and GSSG (0.5–16 μmol/L) were prepared by diluting the stock solutions with 0.5% MPA. All chemicals except boric acid (J.T. Baker) and NaOH (VWR) were purchased from Sigma Aldrich.

### Measurement of blood count, NO metabolites and basal TXB_2_ in plasma, G-CSF and serotonin concentrations in plasma and lung homogenates

Blood count was measured with an animal blood counter scil Vet abc (Horiba Medical, France). Concentrations of NO metabolites (NO_2_^-^ and NO_3_^-^) in plasma were measured with an ENO-20 NOx Analyzer (Eicom Corp., Kyoto, Japan). Basal TXB_2_, G-CSF and serotonin concentrations in plasma were determined with ELISA kits (Enzo Life Sciences, ADI-900-002; Thermo Scientific, EMCSF3 and Enzo, ADI-900-175, respectively). For determination of G-CSF and serotonin concentration, lung samples were homogenised in the buffer as previously reported [20] with some modifications and concentrations being measured with the commercially available kits listed above and normalised to total protein measured with BCA.

### Measurement of eicosanoid production by LC-MS/MS

#### Sample preparation

Blood samples were diluted with saline (5 times) and stirred in alternating directions in 1ml cuvettes with disposable, siliconised stir bars (Chrono-Log, US) for one hour at 37°C (1,500 rpm; spinning time in one direction: 3 s; acceleration/deceleration: 20,000 rpm·s^-1^) in a specially designed Xyzyk apparatus (Xyzyk Co, Poland), as described previously [21]. To determine the generation of eicosanoids *ex vivo* in the stirred blood, samples were taken on aspirin (500 μM) after 60 minutes of *ex vivo* stirring in Xyzyk apparatus and centrifuged to obtain plasma (3000xg, 12 min, 4°C). All plasma samples were stored at -80°C for further eicosanoid analysis including TXB_2_, 6-keto-PGF_1α_, PGE_2_, PGD_2_, PGF_2α_, 8,9-, 11,12- and 14,15-EETs, 5-, 12-, 15-, 19- and 20-HETEs.

Urine samples after collection were clarified by centrifugation and kept at -80°C for analysis of TXA_2_ stable metabolite (2,3-dinor TXB_2_). The concentration of 2,3-dinor TXB_2_ was normalised to creatinine level.

Plasma and urine samples were prepared for the LC-MS/MS assay according to the protocol slightly changed from that previously described in [22, 23]. Briefly, after the addition of internal standard mixture, all samples were purified applying liquid-liquid extraction using acidified ethyl acetate. After the evaporation of organic layer, the dry residues were reconstituted in ethanol and samples were directly injected into LC-MS/MS system.

Calibration and quality control samples were prepared using artificial plasma or urine [22, 23] spiked with known concentration of eicosanoid working standard solutions and extracted according to the same procedure applied for the biological samples.

#### LC-MS/MS conditions and eicosanoid quantification

The LC-MS/MS system applied for eicosanoid quantification consisted of a UFLC Nexera liquid chromatograph (Shimadzu, Kyoto, Japan) and triple-quadrupole mass spectrometer Qtrap 5500 (Sciex, Framingham, Maryland, USA) equipped with electrospray ion source.

The chromatographic separation of eicosanoids was achieved applying an Acquity UPLC BEH C18 (3.0x100 mm, 1.7 μm, Waters, Milford, Massachusetts, USA) analytical column. The mobile phases were delivered in gradient elution mode and consisted of 0.1% FA in ACN and 0.1% FA in H_2_O for plasma sample analysis and ACN and H_2_O+0.1%NH_4_OH for urine sample assay. The data acquisition was carried out in multiple reaction monitoring mode (MRM) in the negative polarisation mode for all eicosanoids (TXB_2_, 6-keto-PGF_1α_, PGE_2_, PGD_2_, PGF_2α_, 8,9-, 11,12- and 14,15-EETs, 5-, 12-, 15-, 19- and 20-HETEs, 2,3-dinor TXB_2_) and their deuterated internal standards (TXB_2_-d_4_, 6-keto-PGF_1α_-d_4_, PGE_2_-d_4_, PGD_2_-d_4_, PGF_2α_-d_4_, 14,15-EET-d_11_, 5-HETE-d_8_, 12-HETE-d_8_, 15-HETE-d_8_, 20-HETE-d_6_, 2,3-dinor TXB_2_-d_9_).

The most specific and abundant ion transitions (Q1→Q3) for all measured eicosanoids and their internal standards were thoroughly selected and used for quantification. The calibration curves were plotted as the relationship between the peak area ratios of analyte/internal standard to the nominal concentration of the analyte. Eicosanoid levels in plasma and urine samples were calculated based on the regression equations estimated for each analyte. The LC-MS/MS-based methods applied for eicosanoid quantification were validated and previously described [22, 23].

Eicosanoid standards and their deuterated internal standards were bought from Cayman Chemical.

### Measurement of platelet activation by flow cytometry

Blood samples were diluted with saline and washed with Tyrode buffer. Each sample was double stained with four-antibody panels from Emfret Analytics used at final dilution of 1:8. All staining panels included platelet-specific antigen GpIIbIIIa (CD41/61), either FITC (cat no M025-1, monoclonal (clone Leo.F2), rat (Wistar) IgG2a) or PE (cat no M025-2, monoclonal (clone Leo.F2), rat (Wistar) IgG2a)-conjugated, for platelet identification and one of four platelet activation markers: PE-conjugated active form of GPIIb/IIIa (cat no M023-2, monoclonal (clone JON/A), rat IgG2b) and P-selectin (cat no M130-2, monoclonal (clone Wug.E9), rat (Wistar) IgG1) antibodies; FITC-conjugated fibrinogen (cat no P140-1, rabbit polyclonal IgG) or von Willebrand (vWF) factor–representing platelet binding capacity (cat no P150-1, rabbit polyclonal IgG). Platelets were identified based on their forward- and side-scatter characteristics and were gated on the basis of the expression of platelet-specific antigen CD41/61 as shown previously [16]. Isotype control antibodies either FITC- (cat no P190-1, rat polyclonal IgG) or PE (cat no P190-2, rat polyclonal IgG) -conjugated were used to assess non-specific binding for each individual sample. Basal, ADP (20 μM) and thrombin (0.025 U·ml^-1^)-induced activation of platelets was assessed on the basis of the measured expressions/binding level of surface membrane antigens expressed as the percentage of all platelets above the isotype control fluorescent signal and the median fluorescence intensity (MFI). Flow cytometric analyses of platelet activation was performed using flow cytometry software (LSRII and FACS/Diva ver. 6.0, respectively, Becton Dickinson, Oxford, UK). Measurements were made on a logarithmic scale and at least 10 000 events were collected. Appropriate colour compensation was determined in samples singly stained with either FITC-conjugated anti-CD41/61 or PE-conjugated anti-CD41/61.

### Statistical analysis

Statistical significance was assessed in GraphPad Prism 5.03 with a parametric unpaired two-sided T test or non-parametric Mann-Whitney test based on the normality of the data distribution (tested with a Shapiro-Wilk normality test), homogeneity of variances (tested with F test) and the variable scale. The data were presented as mean ± SD or median of the data and interquartile range (IQR) (from lower [25%] to upper [75%] quartile). Only P values ≤0.05 were considered significant.

## Results

### Effects of long-term ASA treatment on systemic eicosanoid production and platelet reactivity in breast cancer-bearing mice

**Fig 1:**
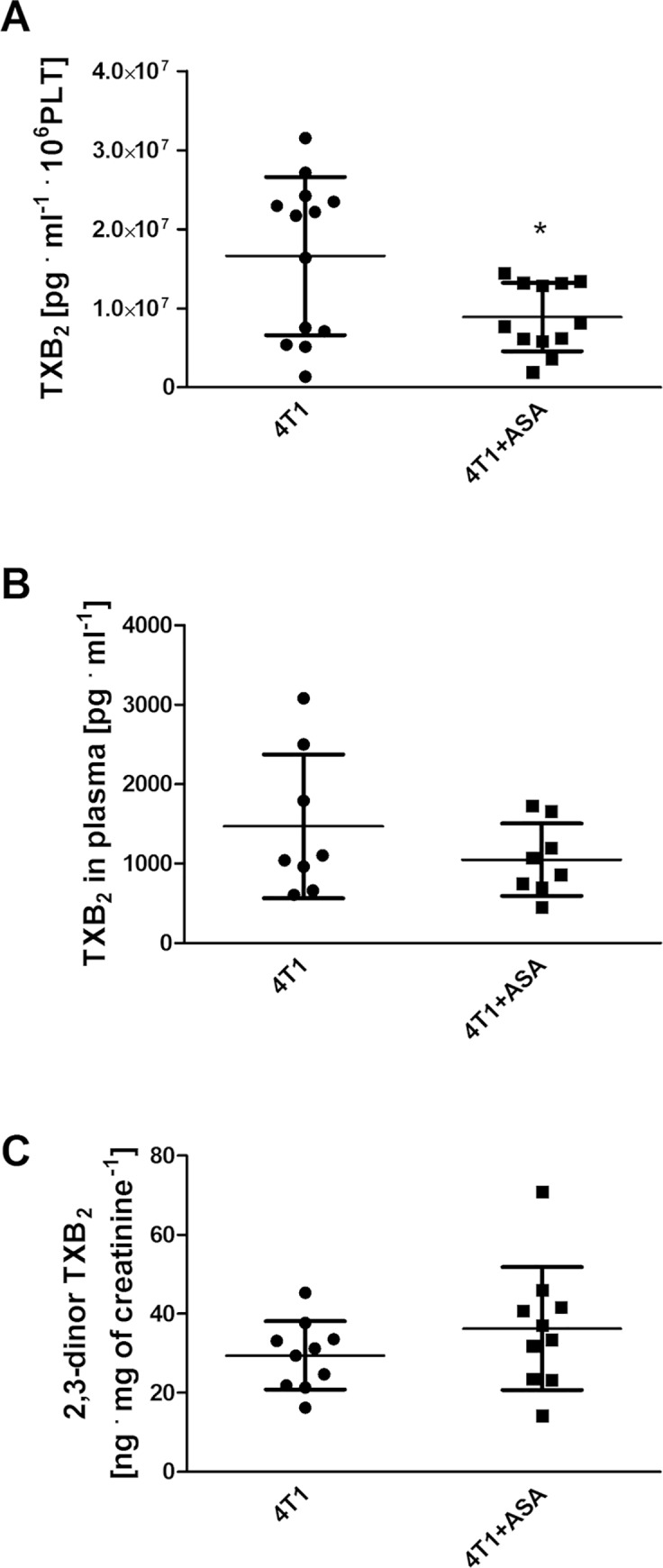
TXB_2_ production in *ex vivo* blood assay. Data are presented as mean ± SD. TXB_2_ (the stable product of TXA_2_ hydrolysis) concentration after stirring of blood samples *ex vivo* was measured by LC-MS/MS (A) as described in *Materials and Methods*. For comparison, basal TXB_2_ concentration in plasma (B) was measured using ELISA kits and the concentration of TXB_2_ metabolite 2,3-dinor-TXB_2_ in urine (C) was determined by LC-MS/MS. The data were analysed with an unpaired two-sided T-test based on their normality of distribution (Shapiro-Wilk test) and equality of variances (F test). In the case of (B), an unpaired T-test with Welch’s correction was used because variances of the groups were not equal. The symbol * denotes statistical significance between 4T1+ ASA vs 4T1 untreated mice at the level of P≤0.05.

**Fig 2:**
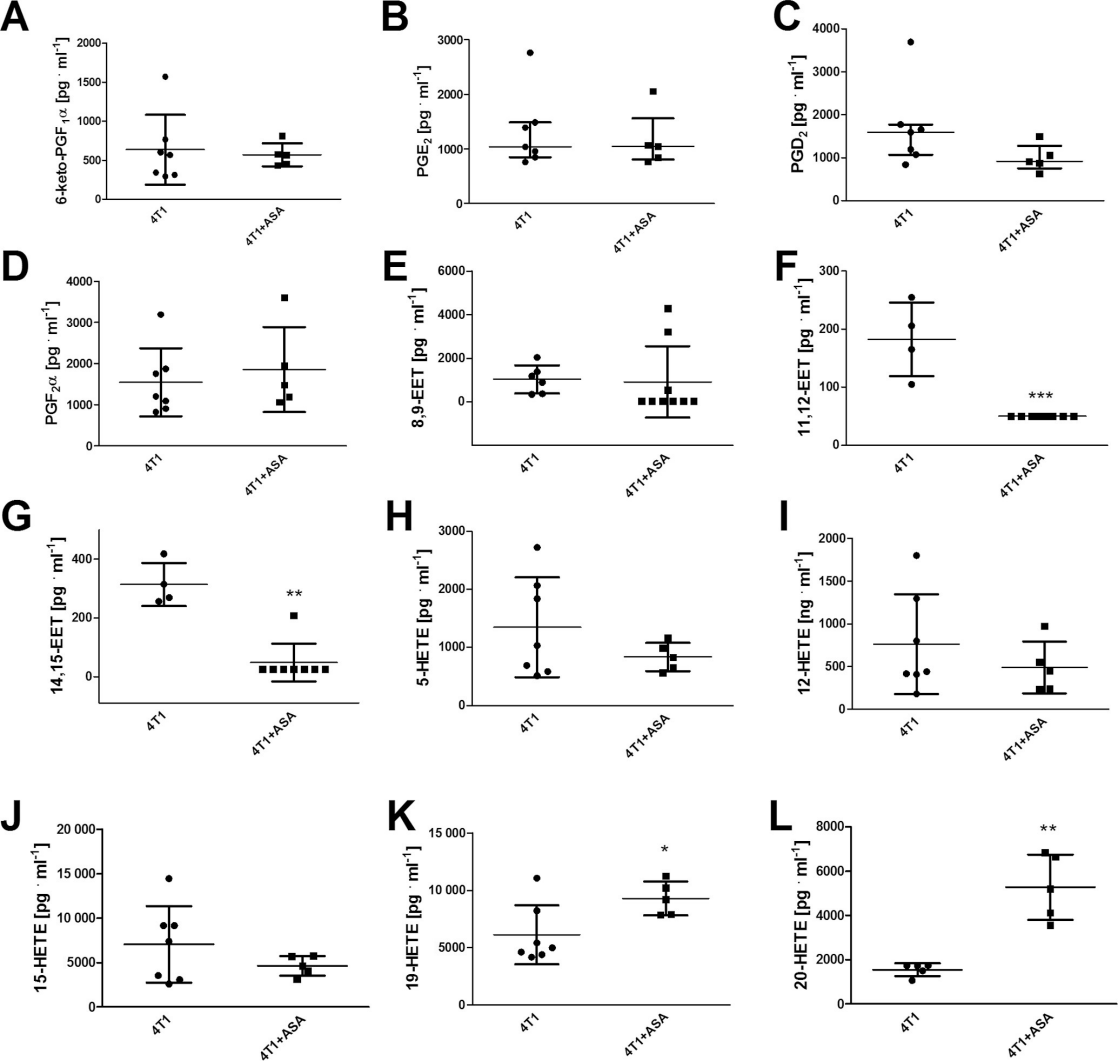
Eicosanoid production in *ex vivo* blood assay. The data are presented as median and IQR; for values <LOQ, the limit value of the method was used. After euthanasia of 4T1+ASA and control 4T1 mice, blood samples were stimulated (stirring) for 1 h as described in *Materials and Methods*. Subsequently, plasma was used for LC-MS/MS quantification of the panel of eicosanoid production: 6-keto PGF_1α_ (6-keto-prostaglandin F_1α_; stable metabolite of prostacyclin (PGI_2_) (**A**), PGE_2_ (prostaglandin E_2_) (**B**), PGD_2_ (prostaglandin D_2_) (**C**), PGF_2α_ (prostaglandin F_2α_) (**D**), 8,9-EET (**E**), 11,12-EET (**F**), 14,15-EET (**G**) (8,9-, 11,12-, and 14, 15-epoxyeicosatrienoic acids), 5-HETE (**H**), 12-HETE (**I**), 15-HETE (**J**), 19-HETE (**K**) and 20-HETE (**L**) (5-, 12-, 15-, 19- and 20-hydroxyeicosatetraenoic acids). The data were analysed with an unpaired two-sided T test (**A, D, H, I, J, K, L**) or non-parametric Mann-Whitney test (**B,C,E,F,G**) based on their normality of distribution (Shapiro-Wilk normality test) and equality of variances (F test). Symbol * denotes statistical significance between 4T1+ASA1 mice and 4T1 untreated animals at the level of P≤0.05 (*), P≤0.01 (**) and P≤0.001 (***).

**Fig 3:**
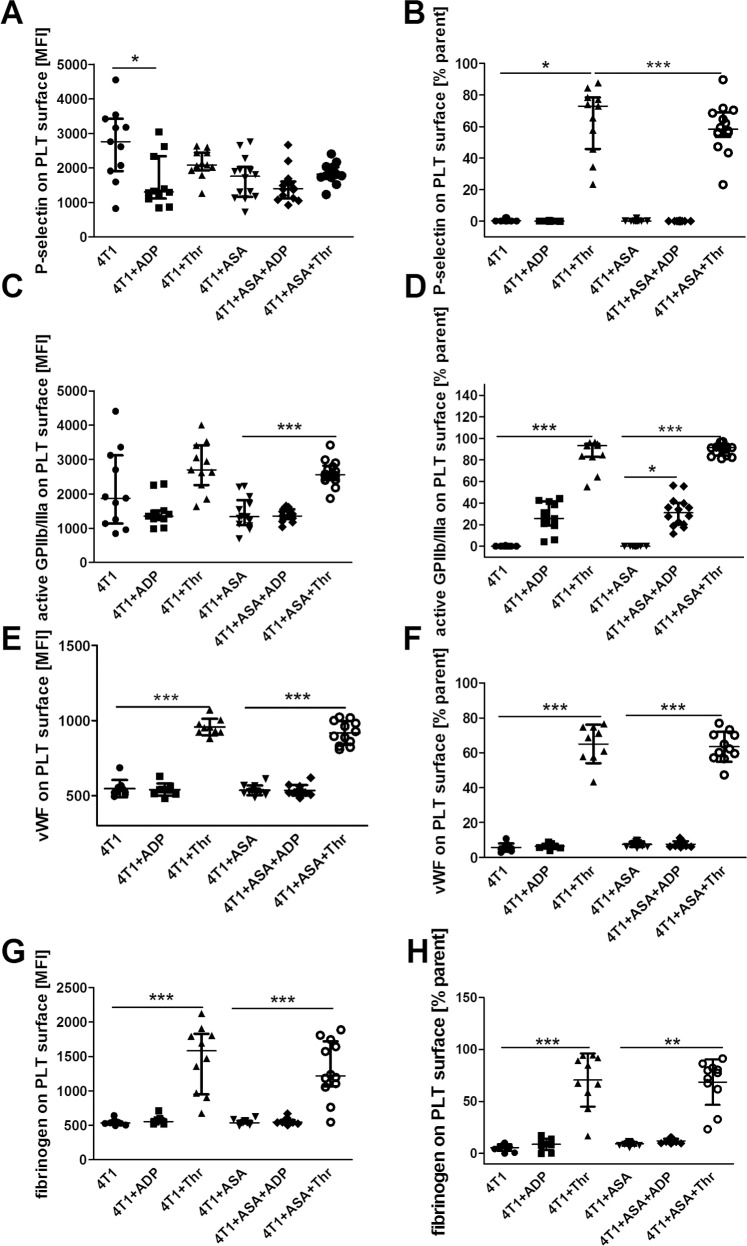
Platelet reactivity to ADP and thrombin. The data are presented as the mean ± SD (**E, F**) or the median and IQR (**A-D** and **G, H**). Platelet basal as well as ADP and thrombin-stimulated surface antigen exposure was measured as described in *Materials and Methods*. The symbol * indicates statistically significant difference between basal and ADP or thrombin-stimulated platelets at the level of P≤0.05 (*), P≤0.01 (**) and P≤0.001 (***), respectively. The data were analysed with parametric One-Way ANOVA followed by Tukey’s post-hoc test or non-parametric Kruskall-Wallis test followed by Dunn’s multiple comparison test based on the normality of distribution (Kolmogorov-Smirnov test) and equality of variances (Barlett’s test).

ASA treatment exerted anti-platelet effects (Fig 1A); however, these effects were only observed in *ex vivo* assays of the TXB_2_ generation in stirred blood (1.7±1.0x10^7^ pg·ml^-1^·10^6^ PLT in the blood of breast 4T1 cancer-bearing mice vs 1.0±0.6x10^7^ pg·ml^-1^·10^6^ PLT in the blood of breast cancer-bearing mice treated with ASA (4T1+ASA), P = 0.0219) (Fig 1A), whereas basal TXB_2_ plasma concentration (Fig 1B) as well as 2,3-dinor-TXB_2_ concentration in urine (Fig 1C) were not changed. The production of other prostanoids (6-keto-PGF_1α_ (stable metabolite of PGI_2_), PGE_2_, PGD_2_ and PGF_2α_) (Fig 2A–2D) in the stirred blood was not altered between 4T1 and 4T1+ASA groups. However, ASA treatment resulted in a substantial decrease of 11,12-EET and 14,15-EET generation, causing a decrease in their concentration below the limit of quantification of the applied LC-MS/MS method in the majority of the samples (185.3; 119.6–242.3 pg·ml^-1^ for 4T1 vs <50.0 pg·ml^-1^ for 4T1+ASA group, P = 0.0010 and 291.7; 259.3–392.1 pg·ml^-1^ for 4T1 vs <25.0 pg·ml^-1^ for 4T1+ASA group, P = 0.0033, respectively) (Fig 2F and 2G). The level of 5-, 12- and 15-HETE was unchanged and independent of ASA administration (Fig 2H–2J), whereas the production of 19-HETE was increased in the stirred blood of ASA-treated cancer-bearing mice (4,980.0; 4,390.0–8200.0 pg·ml^-1^ for 4T1 vs 9,200.0; 7,875.0–10,725.0 pg·ml^-1^ for 4T1+ASA group, P = 0.0335) (Fig 2K) concomitantly with 20-HETE (1,720.0; 1,283.0–1,740.0 pg·ml^-1^ for 4T1 vs 5,200.0; 3,840.0–6,750.0 pg·ml^-1^ for the 4T1+ASA group, P = 0.0052) (Fig 2L). Surprisingly, ASA treatment had little effect on basal, ADP and thrombin-induced PLT reactivity as assessed by flow cytometry (Fig 3A–3H).

### Effects of long-term ASA treatment on breast cancer progression and blood count of cancer-bearing mice

**Fig 4:**
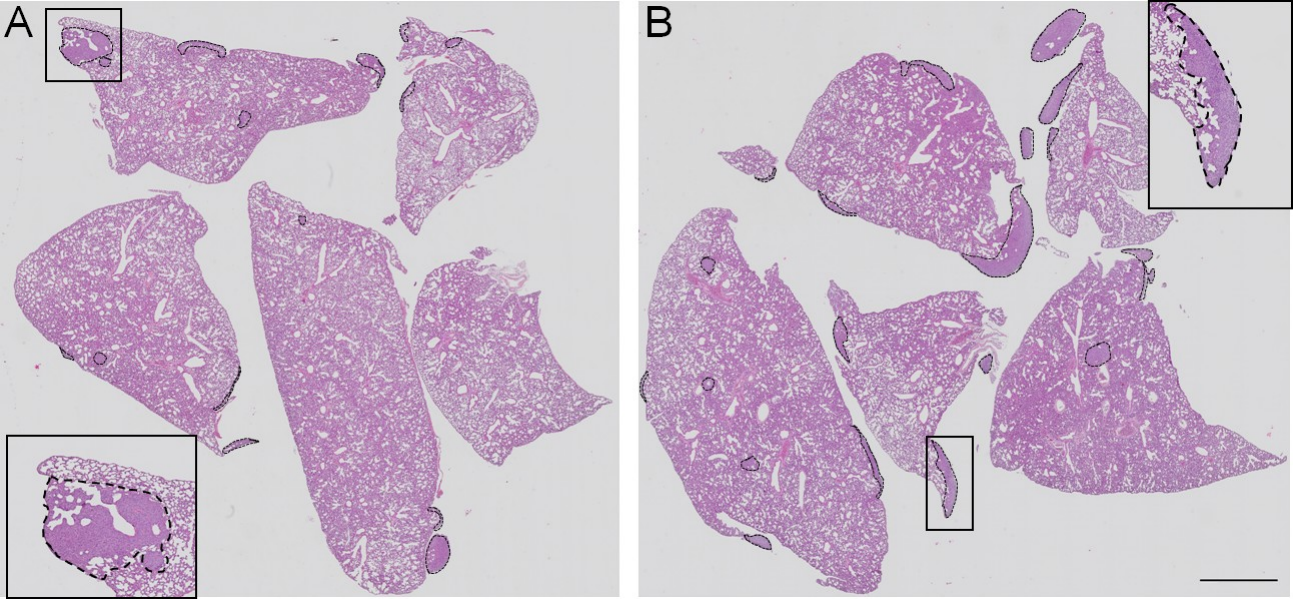
Representative lung histological sections showing pulmonary metastasis in 4T1 and 4T1+ ASA groups of mice. Lung slices were stained with hematoxylin and eosin (H&E) to visualise pulmonary metastasis in untreated 4T1 and ASA-treated (4T1+ASA) groups of mice and scanned with a BX51 microscope equipped with the virtual microscopy system dotSlide (objective magnification 10x; Olympus, Japan). The representative images for 4T1 and 4T1+ASA mice are shown in (**A**) and (**B**), respectively, and pulmonary metastases were indicated with the dotted line. Scale bar represents 2 mm.

The number of pulmonary metastases, relative pulmonary metastatic area and primary tumour weight were not affected in the 4T1+ASA group as compared with the 4T1 group (Table 1). Intriguingly, the weight of the lungs was higher in breast cancer-bearing mice treated with ASA. These results stayed in line with reduced lung airness, increased area occupied by cell nuclei in the lung parenchyma and higher number of circulating granulocytes in response to ASA treatment of cancer-bearing mice (Table 1). There was also an increase in platelet count (PLT) as well as higher absolute neutrophil count (ANC) and absolute neutrophil to absolute lymphocyte ratio (NLR) in mice from the 4T1+ASA group vs the control 4T1 group (Table 1). The representative images of pulmonary metastases in 4T1 and 4T1+ASA group of mice are shown in Fig 4).

**Table 1:** Blood count and pulmonary metastasis in breast cancer-bearing mice.

Parameter	Experimental groups	
	4T1	4T1+ASA
No of pulmonary metastases	36; 27–74 (n = 9)	55; 29–133 (n = 9)
Relative pulmonary metastatic area [%]	9.8±11.5 (n = 9)	16.9±15.1 (n = 9)
Primary tumor weight [% of BW]	9.4±4.8 (n = 22)	11.1±5.5 (n = 23)
Lung weight [% of BW]	1.2; 0.9–1.5 (n = 22)	1.7; 1.2–2.3 (n = 23)*
Airness of lungs [%]	44.4; 33.9–48.6 (n = 90)	32.5; 26.1–56.3 (n = 90)***
Nuclei area in the lungs [%]	28.0; 25.3–31.8 (n = 90)	29.3; 25.9–32.9 (n = 90)*
WBC [K·μl^-1^]	276.7; 154.3–385.1 (n = 13)	348.4; 270.0–468.2 (n = 13)
GRA [K·μl^-1^]	128.7±54.1 (n = 12)	220.7±104.2* (n = 14)
ANC [K·μl^-1^]	136239.0±86715.0 (n = 13)	309004.0±226538.0* (n = 14)
LYM [K·μl^-1^]	89.5±61.1 (n = 13)	73.0±35.2 (n = 14)
ALC [K·μl^-1^]	86573±64807 (n = 13)	103812±78990 (n = 14)
NLR [AU]	1.5; 1.3–2.2 (n = 12)	3.2; 2.2–4.0 (n = 14)**
Circulating RBC [M·μl^-1^]	9.0±0.6 (n = 13)	9.1±0.7 (n = 14)
HGB [g·dl^-1^]	14.3±0.9 (n = 13)	14.4±1.1 (13)
PLT [K·μl^-1^]	812.3±124.9 (n = 13)	1026.0±203.5 (n = 13) **

Data are presented as the mean ± SD or the median and IQR (interquartile range). Low-dose aspirin was administered to mice starting seven days prior to orthotopic inoculation of 4T1 breast cancer cells (4T1+ASA) at a dose of ~ 12 mg per 1 kg of body weight per 24 h and continued until the 5^th^ terminal week of the disease. At that time, mice from 4T1-injected group and the 4T1-injected group treated with ASA were euthanised; blood samples were collected to measure blood count (WBC, GRA, ANC (also known as absolute granulocyte count AGC: WBC x %GRA), LYM, ALC-absolute lymphocyte count (WBC x %LYM); NLR-absolute neutrophil/absolute lymphocyte ratio; RBC, HGB, PLT). Primary tumour and lung weight was presented as percentage of body weight (% of BW). Lungs were excised, weighed, fixed in formalin, cut into lobes and visible metastatic nodules were counted. Subsequently, lung lobes were paraffin-embedded, cut into slices and stained with H&E to measure the relative area of pulmonary metastases. Airness of the lungs and lung nuclei area were measured as described in *Materials and Methods* and presented as the % of the lung cross-section. Symbol * denotes statistical significance between 4T1 and 4T1+ ASA mice at the level of P≤0.05 (*), P≤0.01 (**), P≤0.001 (***). Some data sets were Box-Cox transformed. Based on the normality of distribution assessed with a Shapiro-Wilk normality test, equality of variances was assessed with an F test and the variable scale, and data were analysed with a two-sided unpaired T-test or non-parametric Mann-Whitney test.

### Effects of long-term ASA treatment on G-CSF and serotonin concentrations in breast cancer-bearing mice

**Fig 5:**
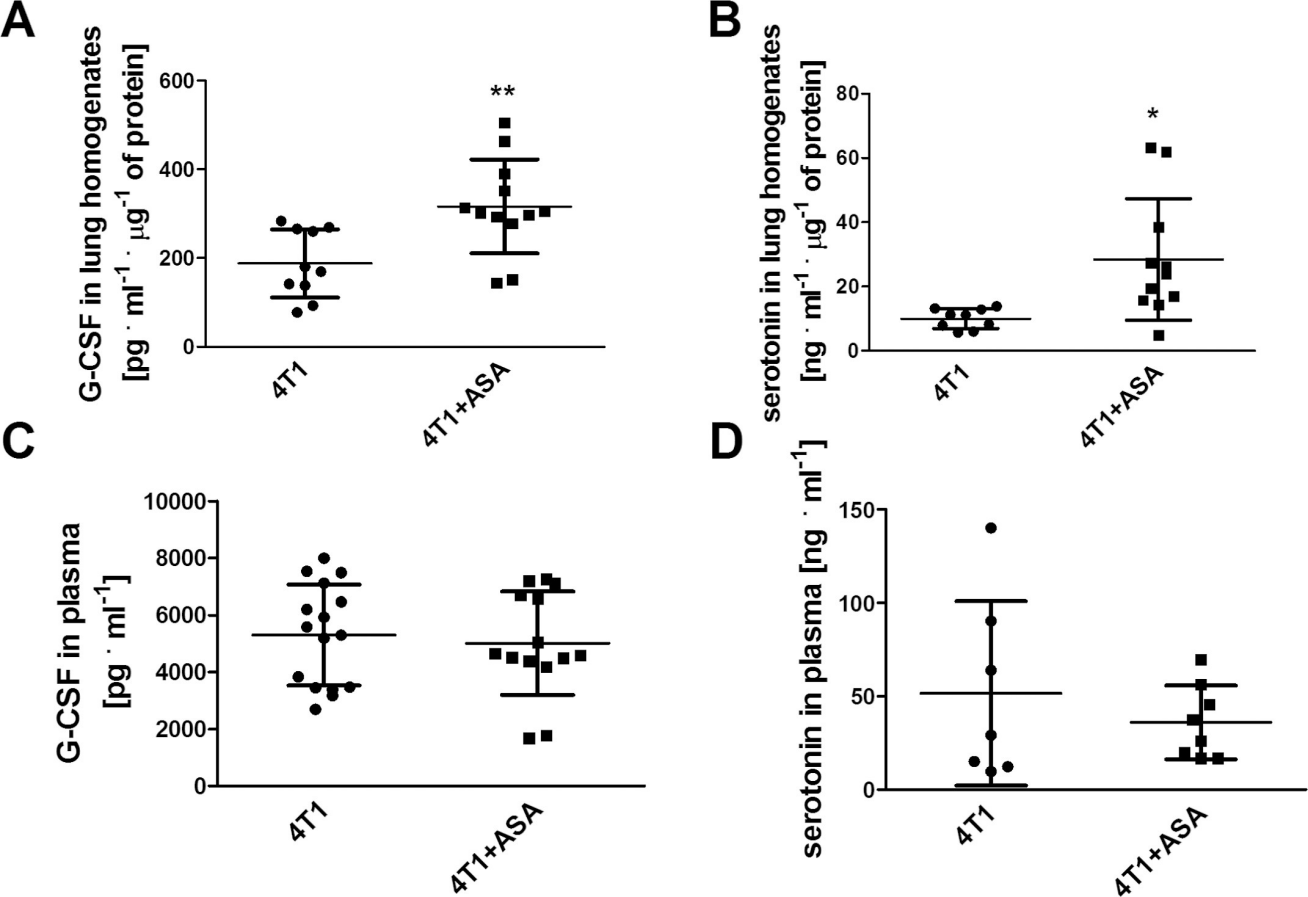
G-CSF and serotonin concentration in lungs and plasma. Data are presented as mean ± SD. ASA was administered to mice starting seven days before orthotopic inoculation of 4T1 breast cancer cells (4T1+ASA) at a dose of ~ 12 mg per 1 kg of body weight per 24 h and was continued until the 5^th^ terminal week of the disease. At that time, mice from both 4T1+ASA and 4T1 control groups were euthanised, and lungs were isolated for measurement of G-CSF (Granulocyte Colony Stimulating Factor) (**A**) and serotonin (**B**) concentrations as well as G-CSF (**C**) and serotonin (**D**) concentrations in the plasma as described in *Materials and Methods*. The data were analysed with a parametric two-sided T-test based on their normality of distribution (Shapiro-Wilk test) and equality of variances (F test). In the case of (**D**), an unpaired T-test with Welch’s correction was used due to the fact that variances of the groups were not equal. The symbols * and ** denote statistical significance between 4T1+ASA vs 4T1 mice at the level of P≤0.05 and P≤0.01, respectively.

In line with more pronounced infiltration of the lung parenchyma of 4T1+ASA group of mice by inflammatory cells as evidenced by increased nuclei area in the lungs (Table 1), the concentration of granulocyte-colony stimulating factor (G-CSF) was increased in lung homogenates of the 4T1+ASA group as compared to the 4T1 group (316.3±106.1 pg·ml^-1^ μg of protein^-1^ vs 187.7±76.9 pg·ml^-1^ μg of protein^-1^, respectively, P = 0.0046) (Fig 5A). Similarly, the concentration of serotonin (related to pro-inflammatory environment in the lungs [24]) was also higher in the lung homogenates of 4T1+ASA mice (28.4±19.0 ng·ml^-1^ μg of protein^-1^ vs 10.0±3.1 ng·ml^-1^ μg of protein^-1^ in 4T1 group, P = 0.0100) (Fig 5B). In contrast to the lungs, there was no difference in G-CSF and serotonin concentrations in the plasma (Fig 5C and 5D).

### Effects of long-term ASA treatment on NO production in the lungs and systemic NO bioavailability in the plasma of breast cancer-bearing mice

**Fig 6:**
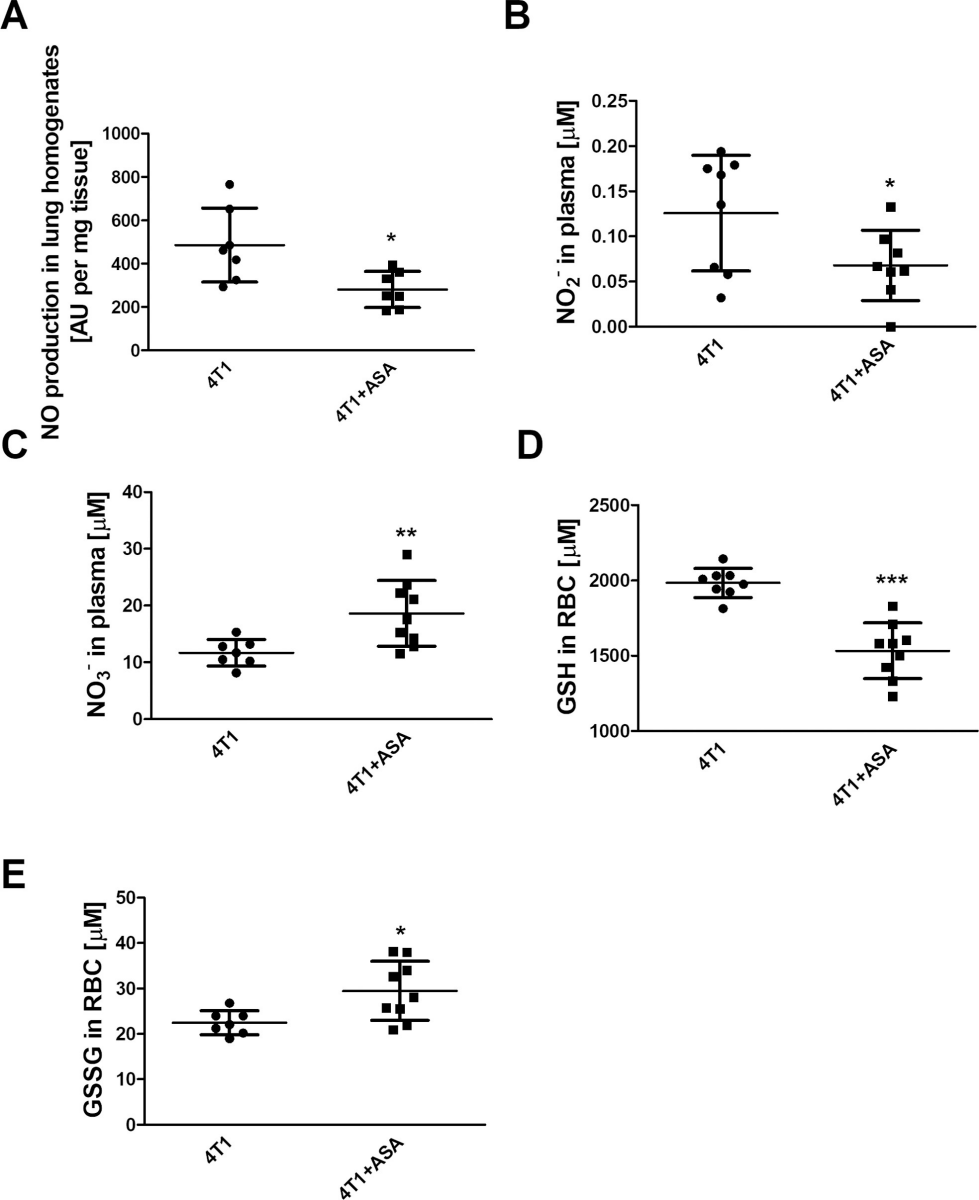
NO production in lungs, systemic NO bioavailability and oxidative status of RBC. Data are presented as the mean ± SD. Lungs of 4T1+ASA and 4T1 mice were collected to measure production of NO with Fe(DETC)_2_ (**A**), whereas systemic NO bioavailability was assessed in the plasma by means of the concentration of NO_2_^-^ (**B**) and NO_3_^-^ (**C**). Concentration of GSH (**D**) and GSSG (**E**) was measured in RBC of breast cancer-bearing mice treated (4T1+ASA) or not (4T1) with ASA as described in *Materials and Methods*. Data were analysed using an unpaired two-sided T-test. Normality of distribution and equality of variances were tested with both Shapiro-Wilk and F tests, respectively, and in the case of (**C**) and (**E**), an unpaired T-test with Welch’s correction was used because variances of the groups were not equal. Symbol * denotes statistical significance between 4T1+ASA vs 4T1 mice at the level of P≤0.05 (*), P≤0.01 (**) and P≤0.001 (***).

In the metastatic lungs of the 4T1+ASA group, production of nitric oxide was decreased as compared to the 4T1 group (280.2±83.2 AU·mg^-1^ of tissue vs 485.8±170.4 AU·mg^-1^ of tissue, respectively, P = 0.0141) (Fig 6A). In the plasma, NO_2_^-^ concentration was lower in ASA-treated mice compared to the 4T1 untreated group (0.068±0.039 μM vs 0.126±0.064 μM, respectively, P = 0.0463) (Fig 6B), whereas NO_3_^-^ concentration was elevated in the 4T1+ASA group as compared to the 4T1 control group (18.62±5.8 μM vs 11.68±2.3 μM, respectively, P = 0.0075) (Fig 6C).

### Effects of long-term ASA treatment on GSH and GSSG concentration in circulating RBC of breast cancer-bearing mice

The concentration of reduced glutathione (GSH) was lower in RBC isolated from the 4T1+ASA group in comparison with the control 4T1 group (1,533.0±184.9 μM vs 1,984.0±96.5 μM, respectively, P<0.0001) (Fig 6D). In turn, the concentration of the oxidised form of glutathione was elevated in breast cancer-bearing mice treated with ASA compared to the 4T1 group (29.5±6.5 μM vs 22.5±2.6 μM, respectively, P = 0.0136) (Fig 6E).

## Discussion

Acetylsalicylic acid (ASA, aspirin), already attracted attention several decades ago with respect to its anti-cancer/anti-metastatic properties [25]. These anti-metastatic effects of low-dose ASA (≤100 mg in humans) can be attributed to the inhibition of COX-1/thromboxane A_2_ (TXA_2_) pathway in platelets [2]. Indeed, platelet COX-1 -derived TXA_2_ has been recently shown to play a central role in the early phase of intravascular metastasis in the models of experimental metastasis and spontaneous metastasis in mice but not in the extravasation or extravascular phases of metastasis [15]. Our study extended results of Lucotti et al. [15] with regard to the effects of ASA on the late phase of metastasis in mice. Long-term treatment with ASA at a low dose did not affect the number of pulmonary metastases in a murine orthotopic model of 4T1 breast cancer in a previous report [15]. In our study, we confirmed lack of the effect of ASA on number of metastasis, but, quite surprisingly, we found that long-term treatment with ASA negatively affected the phenotype of late-phase breast cancer, evidenced by decreased lung airness, higher G-CSF concentrations in the lung homogenates as well as decreased local pulmonary and systemic NO bioavailability along with increased GSSG/lower GSH concentrations in RBC and some unfavourable changes in eicosanoid generation in the blood of mice.

Undoubtedly, it was the most intriguing finding of this study to discover that lungs of breast cancer-bearing mice receiving long-term treatment with ASA were more infiltrated by inflammatory cells when compared with control 4T1 mice that did not receive ASA (please compare nuclei area in the lungs in the Table 1). Concomitantly, lungs of mice bearing breast cancer subjected to long-term treatment with ASA displayed reduced airness and increased weight (Table 1). More pronounced infiltration of lung parenchyma by inflammatory cells in breast cancer-bearing mice receiving long-term treatment with ASA was also accompanied by increased concentration of serotonin in their lung homogenates (Fig 5B) indicating a more pro-inflammatory environment as reported by [24]. Additionally, in the lungs (but not in the plasma) of breast cancer-bearing mice subjected to long-term treatment with ASA, G-CSF production was higher (please compare Fig 5A and 5C). The cytokine G-CSF is known to increase neutrophil survival, potentiate their responses to chemotactic signals [26] and increase immunosuppression due to the possible augmented accumulation of Myeloid-Derived Suppressor Cells (MDSCs) [27, 28], observations which are in accordance with the suggestion that ASA may increase immunotolerance in BALB/c mice [29]. Additionally, treatment with ASA as well as other NSAIDs was shown to potentiate recruitment of haematopoietic stem cells (HSCs) from bone marrow in mice in the presence of G-CSF [30]. More prominent inflammation of pulmonary parenchyma of breast cancer-bearing mice receiving long-term ASA treatment was also reflected by compromised NO production in the lungs (Fig 6A) and systemic reduction of NO bioavailability as evidenced by lower NO_2_^-^ concentration in the plasma (Fig 6B). Given the fact, that NO-dependent function represents key element controlling cancer cell extravasation in the targeted organ [16, 31] and regulates endothelial permeability *in vitro* [32] and *in vivo* [33], these results underscore negative influence of long-term ASA administration to breast cancer-bearing mice on endothelial barrier and NO-dependent vasoprotective mechanisms. Simultaneously with NO deficiency, in circulating RBCs the concentration of reduced glutathione (GSH) was lower (Fig 6D), and the concentration of its oxidised counterpart (GSSG) was higher (Fig 6E). These data indicated more pronounced systemic oxidative stress in ASA-treated breast cancer-bearing mice compatible with a NO-deficiency state.

Negative effects of long-term ASA administration to breast cancer-bearing mice shown in our work (however without effects on number of pulmonary metastasis and the size of primary tumours) stay in contrast with the numerous reports on anti-cancer/anti-metastatic or, at least, not harmful effects of ASA on cancer progression but seem consistent with recent report showing that long-term administration of low-dose aspirin to healthy elderly people increased the number of cancer-related deaths [8].

Previously, Maity et al., [34]) demonstrated that ASA prevented breast tumour cell growth *in vitro*, tumour growth in a nude mice xenograft model and reduced self-renewal capacity and growth of breast tumour-initiating cells. ASA also relieved the metastatic burden to regional lymph nodes in an orthotopic model of lung cancer [35] and compromised the number of secondary tumours after intravenous injection of cancer cells [15, 36]. Furthermore, in a murine model of colorectal cancer (HT29), 20 mg·kg^-1^ of aspirin administered four days before and for one week after cancer cell intravascular injection prevented an increase in the rate of pulmonary metastasis in mice injected with cancer cells previously co-cultured with platelets. In these experimental conditions, ASA profoundly inhibited TXB_2_ concentration in serum and lowered urinary levels of 2,3-dinor-TXB_2_ (a major enzymatic metabolite of TXB_2_) without inhibiting systemic PGI_2_ generation [37], suggesting that the anti-metastatic effect of ASA could be due to inhibition of platelet-derived TXA_2_. In this report, the authors attributed ASA effects also to the inhibition of PGE_2_-dependent mechanisms and platelet-tumour cell interactions. Interestingly, in this study, ASA also did not lower the number of metastasis as compared to mice injected with colon cancer cells alone although lung airness of ASA-treated mice was compromised to a large extent (please see Fig 1A in [37]), similarly as in the present study (Table 1). In fact, higher doses of ASA compared to the one used in our study were required to compromise metastasis in mice, as shown in a recent study by Lucotti et al. [15], and their effectiveness was similar to other pharmacological agents inhibiting COX-1/TXA_2_ pathway in platelets [15]. Most importantly, these anti-metastatic effects of ASA were seen only when applied in the early phase during intravascular metastatic niche formation, but could not be recapitulated in the extravasation or extravascular phases of metastasis, clearly suggesting that the efficacy of ASA to inhibit metastasis was limited to short-term intravascular phase of metastasis.

In our study, we used low-dose ASA (12 mg·kg^-1^ of body weight per day equivalent to ~100 mg per day in humans [1]) for long-term treatment to achieve selective inhibition of COX-1-dependent thromboxane A_2_ generation in platelets without inhibition of COX-1 and COX-2 elsewhere. The treatment with 12 mg·kg^-1^ of ASA decreased platelet-dependent TXB_2_ generation in breast cancer-bearing mice in the *ex vivo* assay of stirred blood (Fig 1A) that allows for highly sensitive assessment of platelet activation, as reported by us previously [21]. However, the inhibitory effect of ASA on TXB_2_ production and platelet reactivity was not detected in other assays (Figs 1B, 1C and 3). Moreover, ASA treatment also affected production of other eicosanoids in the stirred blood because it decreased generation of EETs (known to exert anti-inflammatory and vasoprotective effects [38]) (Fig 2F and 2G), and increased production of 20-HETE (known to promote progression of malignant diseases [39]) and 19-HETE, as shown in Fig 2K and 2L, respectively, without an effect on prostaglandin production (Fig 2A–2D). However, the latter result does not preclude that COX-1 or COX-2 activity could have been locally affected in some organs by ASA treatment because even low-dose ASA could inhibit COX-2 activity in urine as measured by PGE_2_-M (please see S2 Fig in [15]). Accordingly, long-term ASA treatment in mice may exert effects on extra-platelet COX that could have contributed to the detrimental effects of ASA observed in our study. For example, inhibition of COX-dependent PGE_2_ production by ASA in the gut results in gut barrier dysfunction [40, 41] due to dysregulation of the immune response [42]. Similarly, pro-inflammatory effects of ASA could be ascribed to bone marrow where recruitment of haematopoietic stem cells (HSCs) is regulated by COX [30]. Finally, given the recent discovery of the role of platelets in non-classical haemostasis, negative effects of long-term treatment with low-dose ASA could be attributed to impaired endothelial barrier constantly supported by platelets by various mechanisms particularly in the lungs [43–45].

Concluding, ASA administration to cancer-bearing mice induced some pro-inflammatory changes in the profile of eicosanoids compatible with some reports on COX inhibitors [30, 42, 46–48], but in contrast with the general view of ASA as an anti-inflammatory agent inhibiting TXA_2_, 11-HETE and 15-HETE production [49, 50] and stimulating the generation of anti-inflammatory proresolvines (AT-SPMs), such as resolvins (AT-RvDs) and lipoxins (AT-LXs) [50]. Given our findings and those of others, aspirin seems to induce a more complex pattern of changes in the eicosanoid profile in mice as previously thought [50], and, therefore, the effects of low-dose ASA *in vivo* need to be extensively re-evaluated, in particular in inflammatory conditions. These studies should be focused not only on a number of new lipid mediators recently discovered to be affected by aspirin, but also should include COX-independent effects of ASA linked to acetylation of other proteins, RNA and ASA metabolites [51] that may play a role in mice metabolising ASA much faster than humans. To summarise, in our study, the effects of low-dose ASA observed in mice were not equivalent to those in humans because 100 mg per day of ASA in humans inhibits quite selectively COX-1 in platelets and is considered a relatively selective anti-platelet treatment, whereas an equivalent dose in mice (12 mg per kg of body weight) only mildly inhibited the COX-1/TXA_2_ pathway in platelets of breast cancer-bearing mice (Fig 1). Furthermore, such a dose of ASA given to mice also altered the generation of other eicosanoids in cancer-bearing (Fig 2) mice including the inhibition of COX-derived prostanoids far beyond platelet COX-1, inhibition of EET pathway and stimulation of HETE production (5, 19 and 20-HETE) in the blood. To conclude, low-dose aspirin is not a selective murine platelet COX-1/TXA_2_ pathway inhibitor. Altogether, our results demonstrate negative effects of long-term treatment with ASA in a murine orthotopic model of 4T1 breast cancer. Whether these findings could be ascribed to the weakening of endothelial barrier integrity supported by platelets [43, 52] or to other mechanisms remains to be established.
